# RP-HPLC Estimation of Venlafaxine Hydrochloride in Tablet Dosage Forms

**DOI:** 10.4103/0250-474X.40350

**Published:** 2008

**Authors:** S. L. Baldania, K. K. Bhatt, R. S. Mehta, D. A. Shah, Tejal R. Gandhi

**Affiliations:** Department of Pharmaceutical Chemistry, Opp. Town Hall, Anand - 388 001, India; * Anand Pharmacy College, Opp. Town Hall, Anand - 388 001,; 1 A. R. College of Pharmacy, P.Box No. 19, Vallabh Vidyanagar - 388 120, India

**Keywords:** Venlafaxine hydrochloride, carbamazepine, antidepressant, RP-HPLC, validation

## Abstract

A simple, specific, accurate, and precise reverse phase high performance liquid chromatographic method was developed and validated for the estimation of venlafaxine hydrochloride in tablet dosage forms. A Phenomenex Gemini C-18, 5 μm column having 250 × 4.6 mm i.d. in isocratic mode, with mobile phase containing methanol: 0.05 M potassium dihydrogen orthophosphate (70:30, v/v; pH 6.2) was used. The flow rate was 1.0 ml/min and effluents were monitored at 226 nm. Carbamazepine was used as an internal standard. The retention time of venlafaxine hydrochloride and carbamazepine were 3.7 min and 5.3 min, respectively. The method was validated for specificity, linearity, accuracy, precision, limit of quantification, limit of detection, robustness and solution stability. Limit of detection and limit of quantification for estimation of venlafaxine hydrochloride were found to be 100 ng/ml and 300 ng/ml, respectively. Recoveries of venlafaxine hydrochloride in tablet formulations were found to be in the range of 99.02-101.68%. Proposed method was successfully applied for the quantitative determination of venlafaxine hydrochloride in tablet dosage forms.

Venlafaxine is a bicyclic antidepressant, and is usually categorized as a serotonin-norepinephrine reuptake inhibitor (SNRI), but it has been referred to as a serotonin-norepinephrine-dopamine reuptake inhibitor. Venlafaxine hydrochloride is designated (R/S)-1-[2-(dimethylamino)-1-(4 methoxyphenyl) ethyl] cyclohexanol hydrochloride or ( ± )-1-[a [α- (dimethylamino) methyl] p-methoxybenzyl] cyclohexanol hydrochloride salt and has the empirical formula of C_17_H_27_NO_2_. HCl.

Various methods have been reported for estimation of venlafaxine hydrochloride in biological matrices such as plasma, which includes the use of LC with UV detection^1^, LC with electrospray ionization mass spectrometry^2^, LC with coulometric detection^3^, LC with fluorimetric detection^4^,^5^, LC with diode array detection^6^,^7^, GC-MS^8^, LC-MS-MS^9^ and for estimation of it in serum by use of LC^10^. Stability indicating methods have also been reported for its invitro determination in gastric and intestinal fluids^11^ and pharmaceutical formulations^12^.

Both the reported stability indicating methods uses acetonitrile and buffer in various proportions for quantification of venlafaxine hydrochloride. Present study involves development of RP-HPLC method using simple mobile phase containing methanol and buffer for quantitative estimation of venlafaxine hydrochloride in tablet dosage forms which is sensitive and requires shorter analysis time. The developed method was validated as per ICH guidelines^13^,^14^.

The Liquid chromatographic system consisted of following components: Shimadzu HPLC model (VP series) containing LC-10AT (VP series) pump, Variable wavelength programmable UV/VIS detector SPD-10AVP and Rheodyne injector (7725i) with 20 μL fixed loop. Chromatographic analysis was performed using Spinchrom software on a Phenomenex Gemini C18 column with 250 × 4.6 mm i.d. and 5 μm particle size. The Shimadzu electronic balance (AX 200) was used for weighing purpose. Pure samples of venlafaxine hydrochloride and carbamazepine were obtained from Torrent Research Centre, Gandhinagar, Gujarat. Methanol (HPLC grade, purity 99.9%) was procured from E. Merck (India). HPLC grade water was obtained by double distillation and purification through milli-Q water purification system. Potassium dihydrogen orthophosphate (AR grade, purity 99.5%) was procured from Qualigens. Tablet formulation A (Ventab XL, Intas Pharmaceutical Ltd., India) and B (Venlift, Torrent Pharmaceutical Ltd, India) containing labeled amount 42.37 mg venlafaxine hydrochloride (equivalent to 37.5 mg of venlafaxine) were procured from local market.

Potassium dihydrogen phosphate was weighed (2.04 g) and dissolved in 300 ml of water. This solution was mixed with 700 ml of methanol. The solution was sonicated for 10 min and filtered using Whatman filter paper No.1. A stock solution of venlafaxine hydrochloride was prepared by accurately weighing 25 mg of drug, transferring to 25 ml volumetric flask, dissolving in 5 ml of methanol and diluting it upto mark with methanol. Appropriate aliquot of this solution was further diluted with 10 ml of methanol to obtain final standard solution of 50 μg/ml of venlafaxine hydrochloride. Resultant solution was filtered through Whatman filter paper and then used.

A stock solution of carbamazepine was prepared by accurately weighing 25 mg of the drug, transferring to 25 ml volumetric flask, dissolving in 5 ml of methanol and diluting it upto mark with methanol. Appropriate aliquot of this solution was further diluted with 10 ml of methanol to obtain final standard solution of 100 μg/ml of carbamazepine. Resultant solution was filtered through Whatman filter paper and then used.

Twenty tablets were accurately weighed and finely powdered. Tablet powder equivalent to 25 mg of venlafaxine hydrochloride was taken in 25 ml of volumetric flask, resultant solution was filtered through Whatman filter paper and finally volume made upto the mark with same solvent. One millilitre of filtrate was taken in 10 ml volumetric flask and volume was made with methanol upto the mark to obtain concentration of 100 μg/ml. Further 0.1 ml of this solution was taken in 10 ml of volumetric flask. To the same mixture 1 ml of standard carbamazepine stock solution was added and finally diluted to l0 ml with mobile phase to obtain final concentration of 1 μg/ml of venlafaxine hydrochloride and 10 μg/ml of carbamazepine, respectively. The resulting solution was again filtered using Whatman filter paper No. 1 and then was sonicated for 10 min.

A reverse phase C-18 column equilibrated with mobile phase methanol:0.05 M potassium dihydrogen orthophosphate (70:30, v/v; pH 6.2) was used. Mobile phase was filtered through Whatman filter paper and degassed. Mobile phase flow rate was maintained at 1 ml/min and effluents were monitored at 226 nm. The sample was injected using a 20 μL fixed loop, and the total run time was 10 min.

Appropriate aliquots of standard venlafaxine hydrochloride stock solution (0.5 μg/ml) were taken in different 10 ml volumetric flasks, followed by addition of 1 ml of standard carbamazepine standard solution (100 μg/ml) and resultant solutions were diluted up to the mark with mobile phase to obtain final concentration of 0.3, 1, 3, 5, 7, and 9 μg/ml of venlafaxine hydrochloride and 10 μg/ml of carbamazepine, respectively. These solutions were injected into chromatographic system and chromatograms were developed and peak area ratio was determined for each concentration of drug solution. Calibration curve of venlafaxine hydrochloride was constructed by plotting peak area ratio vs applied concentration of venlafaxine hydrochloride and regression equation was computed. Similarly the sample solution was chromatographed and concentrations of venlafaxine hydrochloride in tablet samples were found out using regression equation.

The method was validated for specificity, linearity, accuracy, precision, limit of detection, limit of quantification, robustness and solution stability. Commonly used excipients, such as starch (15%), microcrystalline cellulose (10%), magnesium stearate (1%) and lactose (64%) were spiked into a pre weighed quantity of drug. The chromatograms were taken by appropriate dilutions and the quantities of drugs were determined.

The linearity of the method was determined at the six concentration levels ranging from 0.3-9 μg/ml. The accuracy of the method was determined by calculating recovery of venlafaxine hydrochloride by method of standard addition. Known amount of venlafaxine hydrochloride (0.5, 4, 8 μg/ml) was added to a pre quantified sample solution. The recovery studies were carried out three times over a specified concentration range and the amount of venlafaxine hydrochloride was estimated by measuring the peak area ratios by fitting these values to the straight-line equation of calibration curve. From above determination, percentage recovery and standard deviation of percentage recovery were calculated.

The intra day and inter day precision study was carried out by estimating the corresponding responses 3 times on the same day and on 3 different days (1^st^, 3^rd^ and 5^th^ day) for 3 different concentrations and three repeated injections of venlafaxine hydrochloride (0.5, 2, 7 μg/ml) and the results are reported in terms of relative standard deviation (RSD, Table 2). Repeatability studies were carried out by estimating response of 3 different concentrations of venlafaxine hydrochloride (0.5, 2, 7 μg/ml) for three replicate determinations and results are reported in terms of relative standard deviation (RSD, Table 2).

**TABLE 2:** SUMMARY OF VALIDATION PARAMETERS

Parameters	Values
Detection limit (μg/ml)	0.100
Quantitation limit (μg/ml)	0.300
Accuracy (%)	99.02-101.68
Precision (RSD^a^, %)	
Intraday (*n* = 3)	0.66-2.97
Interday (*n* = 3)	0.38-1.44
Repeatability (RSD^a^, *n* = 3)	0.44-0.92

a RSD indicates relative standard deviation

A calibration curve was prepared using concentrations in the range of 0.1-0.5 μg/ml (expected detection limit range). The standard deviation of y-intercepts of regression line was determined and kept in following equation for the determination of detection limit and quantitation limit. Detection limit = 3.3σ/s; Quantitation limit = 10σ/s, where σ is the standard deviation of y-intercepts of regression lines and s is the slope of the calibration curve.

Robustness of the method was studied by changing the composition of organic phase by ± 5% and the pH by ± 0.2, and also by observing the stability of the drugs for 24 h at 35° temperature in the mobile phase. In order to demonstrate the stability of both standard and sample solutions during analysis, both the solutions were analyzed over a period of 8 h at room temperature. The results showed that for the solutions, retention time and peak area of venlafaxine hydrochloride and internal standard remained almost unchanged and no significant degradation was observed within the indicated period.

UV overlain spectra of both venlafaxine hydrochloride and carbamazepine showed that both the drugs absorbs appreciably at 226 nm, so 226 nm was selected as the detection wavelength in liquid chromatography. Optimization of mobile phase was performed based on resolution, asymmetric factor and peak area obtained. Different mobile phases were tried but satisfactory separation, well resolved and good symmetrical peaks were obtained with the mobile phase methanol: 0.05 M potassium dihydrogen orthophosphate (70:30, v/v; pH 6.2). The retention time of venlafaxine hydrochloride was found to be 3.7 min and that of carbamazepine was found to be 5.3 min respectively (fig. 1). Resolution between venlafaxine hydrochloride and carbamazepine was found to be 5.06, which indicates good separation of both the compounds. Asymmetric factor for venlafaxine hydrochloride was 1.29. Calibration curve for venlafaxine hydrochloride was obtained by plotting the peak area ratio versus the concentration of venlafaxine hydrochloride over the range of 0.3-9 μg/ml, slope and intercept value for calibration curve was y = 0.0414× + 0.00956, and it was found to be linear over entire calibration range studied with r^2^ value of 0.999. The data of regression analysis of the calibration curves are shown in Table 3. Detection limit for venlafaxine hydrochloride was 100 ng/ml and quantitation limit for venlafaxine hydrochloride was 300 ng/ml, which suggest that a nanogram quantity of it can be estimated accurately and precisely. The validation parameters are summarized in Table 2. Recovery of venlafaxine hydrochloride was found to be in the range of 99.02-101.68%. System suitability test parameters are shown in Table 4. Proposed liquid chromatographic method was applied for the estimation of venlafaxine hydrochloride in tablet formulations (Tablet formulation A and B). The result for venlafaxine hydrochloride was comparable with the corresponding labeled amount (Table 1).

**Fig. 1:**
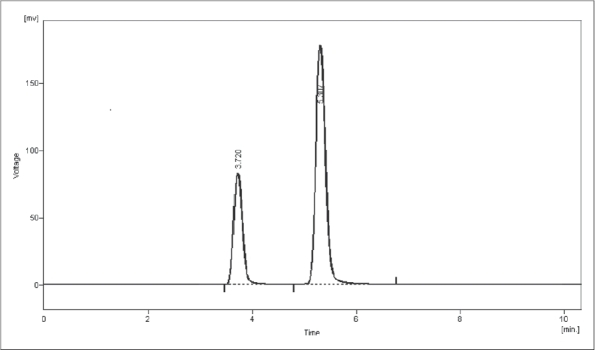
HPLC chromatogram of venlafaxine hydrochloride (RT 3.7 min) and carbamazepine (RT 5.3 min). HPLC chromatogram showing well resolved peaks of venlafaxine hydrochloride and carbamazepine on C-18 Phenomenex Gemini column using methanol: 0.05 M potassium dihydrogen orthophosphate (70:30, v/v; pH 6.2) as mobile phase.

**TABLE 1:** ASSAY RESULTS OF TABLET FORMULATIONS USING PROPOSED METHOD

Formulations	Labeled amount (mg)	Amount obtained (mg)^b^	% Recovery^b^
A	42.37	42.15 ± 0.94	99.49 ± 0.94
B	42.37	43.08 ± 0.91	101.68 ± 0.91

b mean value ± standard deviation of three determinations; Tablet formulation A: (Ventab XL, Intas Pharmaceutical Ltd., India) and B (Venlift, Torrent Pharmaceutical Ltd, India) containing labeled amount 42.37 mg Venlafaxine hydrochloride (equivalent to 37.5 mg of Venlafaxine)

**TABLE 3:** REGRESSION ANALYSIS OF THE CALIBRATION CURVES FOR THE PROPOSED METHOD

Parameters	Values
Calibration range	0.3-9 μg/ml
Slope	0.04134
Standard deviation of slope	0.000114
Intercept	0.00956
Standard deviation of intercept	0.003039
Correlation coefficient (r)	0.9992

**TABLE 4:** SYSTEM SUITABILITY TEST PARAMETERS FOR VENLAFAXINE HYDROCHLORIDE BY THE PROPOSED METHOD

System suitability parameters	Values
Retention time (minute)	3.7
Resolution	5.06
Tailing factor (asymmetric factor)	1.29

Proposed study describes new RP-HPLC method using simple mobile phase for the estimation of venlafaxine hydrochloride in tablet formulations. The method was validated and found to be simple, sensitive, accurate and precise. Percentage of recovery shows that the method is free from interference of the excipients used in the formulation. Therefore the proposed method can be used for routine analysis for estimation of venlafaxine hydrochloride in its tablet formulations.
